# Impact of a Large Fire and Subsequent Pollution Control Failure at a Coke Works on Acute Asthma Exacerbations in Nearby Adult Residents

**DOI:** 10.3390/toxics9070147

**Published:** 2021-06-25

**Authors:** Tricia L. Morphew, Arvind Venkat, John Graham, Matthew Mehalik, Norman Anderson, Deborah Gentile

**Affiliations:** 1 Morphew Consulting LLC, Bothell, WA 98021, USA; tricia@morphewconsulting.com; 2 Allegheny Health Network, Pittsburgh, PA 15212, USA; arvind.venkat@ahn.org; 3 Clean Air Task Force, San Rafael, CA 94901, USA; jgraham@catf.us; 4 Breathe Project, Pittsburgh, PA 15219, USA; mmehalik@breatheproject.org; 5 Anderson Environmental Health LLC, Winslow, ME 04901, USA; andersonenvironmentalhealth@gmail.com; 6 Community Partners in Asthma Care, McMurray, PA 15317, USA

**Keywords:** asthma, outdoor air pollution, particulate matter, sulfur dioxide, hydrogen sulfide

## Abstract

Clairton, Pennsylvania, is home to the largest coke works facility in the United States (US). On 24 December 2018, a large fire occurred at this facility and damaged pollution control equipment. Although repairs were not completed for several months, production continued at pre-fire capacity and daily emissions increased by 24 to 35 times, with multiple exceedances of monitored levels of outdoor air pollution (OAP). The aim of this study was to objectively evaluate the impact of this industrial incident and resultant OAP exceedances on asthma morbidity. We assessed pre-fire and post-fire rate ratios (RR) of outpatient and emergency department (ED) visits for asthma exacerbations among nearby adult residents. Pre-fire versus post-fire RRs increased for both visit types: RR = 1.82 (95% CI: 1.30, 2.53; *p* < 0.001) and 1.84 (95% CI: 1.05, 3.22; *p* = 0.032) for outpatient and ED visits, respectively. Additionally, total visit rates increased on days with OAP exceedances: RR = 2.47 (95% CI: 1.52, 4.01; *p* < 0.0001), 1.58 (95% CI: 1.00, 2.48; *p* = 0.048) and 1.79 (95% CI: 1.27, 2.54; *p* = 0.001) for PM_2.5_, SO_2_, and H_2_S exceedance days, respectively. These results show a near doubling of acute visits for asthma exacerbations in nearby adult residents during this industrial incident and underscore the need for prompt remediation and public notification of OAP exceedances to prevent adverse health impacts.

## 1. Introduction

Clairton, Pennsylvania (PA), is home to the largest coke works facility in the United States (US). On 24 December 2018, a large fire occurred at this facility, which resulted in damage to its desulfurization pollution control equipment. Although repairs to this equipment were not completed for several months, production continued at pre-fire capacity and multiple exceedances of sulfur dioxide (SO_2_), particulate matter less than 2.5 microns in diameter (PM_2.5_) and hydrogen sulfide (H_2_S) occurred during this time. It was estimated that 4685 tons of SO_2_ were released into the environment during this period [1], which is nearly as much SO_2_ emitted annually by all sources in Allegheny County, PA, US [2]. Production was not curtailed since the facility attempted to mitigate outdoor air pollution (OAP) emissions by diluting coke oven gases with natural gas and diverting gases to its other facilities. Initially, it was determined that such efforts might be effective in preventing OAP exceedances; however, approximately five weeks after the fire, OAP exceedances occurred on three consecutive days. This prompted a review of the emission data and the subsequent issuance of an air quality enforcement order on 28 February 2019 [1]. This enforcement order required the facility to comply with emission reductions within 30 days; however, the facility appealed this decision and the new deadline for compliance extended past the repair date.

At the time of the fire and subsequent OAP exceedances, approximately 130,000 people lived within a 5-mile radius of the coke works facility [3]. The first public alert to impacted residents was released 16 days after the fire [4]. Factors contributing to this delay included the initial assessment that OAP mitigation efforts would be successful, and an early report that asthma morbidity, as assessed by chief complaints upon presentation to emergency departments (EDs), was not impacted by this event. However, there were several limitations to this analysis, including reliance on chief complaints at presentation versus specific discharge diagnoses, as well as the short timeframe of assessment, which included only the first few weeks of the industrial incident. Additionally, the analysis captured only ED visits and did not assess acute visits to outpatient clinics. This is important because asthma is recognized as an outpatient disease with most morbidity occurring in the outpatient setting [5].

Recently, a retrospective survey documented increased self-reported asthma symptoms and rescue medication use in adults residing near the facility in the months after the fire [6]. However, that study did not examine the impact on objective measurements such as acute outpatient and ED visits for asthma exacerbations. Additionally, that study examined only SO_2_ emissions and exceedances and did not investigate the impact of confounding factors such as weather inversions and respiratory infections. As such, the aim of this study was to assess the impact of this industrial incident and resultant OAP exceedances, including SO_2_, PM_2.5_, and H_2_S, on the rate of outpatient and ED visits for asthma exacerbations among nearby adult residents. It is acknowledged that this research is not novel; however, such an assessment is important to identify and understand the effect of this event on health outcomes and to guide the development of relevant public policies to protect the health of impacted residents.

The remainder of this article is organized into Section 2, Section 3, Section 4, Section 5 and Section 6 that correspond to literature review, methods, results, discussion, and conclusions, respectively. Section 2 (literature review) summarizes relevant peer-reviewed studies documenting both short-term effects of OAP on respiratory and asthma outcomes, as well as public health efforts to notify and protect impacted residents during industrial incidents. Section 3 (methods) describes data collection and statistical analysis, including outpatient and ED visits for asthma exacerbations, exceedances of pollution emissions, OAP monitor exceedances, weather, and influenza season severity. Section 4 (results) details the results of the study, including population demographics, rates of outpatient and ED visits for asthma exacerbations, pollution emission exceedances, OAP monitor exceedances, weather, and severity of influenza season. Section 5 (discussion) summarizes the study and its results, discusses the significance of the results in relation to other studies, and addresses study strengths and limitations. Section 6 (conclusions) presents recommendations for implementation of specific strategies to better protect health impacts in nearby residents during future events.

## 2. Literature Review

Short-term exposure to OAP, including PM_2.5_, and particularly SO_2_, which is a precursor to sulfate PM_2.5_, has been associated with increased asthma morbidity [7,8,9,10,11,12,13,14]. Mechanistic studies have demonstrated changes in respiratory symptoms and lung function in patients with asthma following exposure to SO_2_ for a duration as short as 10 min [7,8]. Epidemiologic studies have reported increased outpatient and ED visits and hospital admissions following short-term exposure to elevated levels of PM_2.5_ and SO_2_ [9,10,11,12,13,14].

Previous studies have documented short-term respiratory morbidity among patients with asthma during acute exposure to high levels of OAP following industrial incidents. One recent study reported increased asthma related ED visits and hospital admissions during PM_2.5_ elevations caused by a six-week long coal mine fire in Australia [15]. Other studies by that group documented increased symptoms, outpatient visits, and medication use for respiratory disease, including asthma, during this same OAP incident [16,17]. Increased respiratory symptoms were reported by nearby residents during and for several weeks after a large industrial fire in Texas, US, which burned and released PM_2.5_ and black carbon into the atmosphere for several days [18]. A recent meta-analysis confirmed reports of an increased incidence of asthma in first responders involved in rescue and recovery following the attack and fire on the World Trade Center in the US [19]. Other studies have reported increased respiratory morbidity due to other biomass combustion sources, including wildfires and prescribed fires [20,21,22].

In addition to regulating air quality standards to protect public health, recent efforts have focused on providing prompt notification of acute deteriorations in air quality to impacted residents [23,24]. Interestingly, a Canadian asthma monitoring system incorporated simultaneous modeling of health impacts with OAP data [25]. Numerous studies have shown that air quality alerts lead to increased individual behavior changes aimed at reducing OAP exposure [26,27,28,29]. Recent efforts have also focused on building emergency response systems for both natural disasters and unnatural incidents [30,31,32,33]. Many of these efforts incorporate proactive communication, monitoring, and management of environmental risks to protect the health of impacted residents.

## 3. Materials and Methods

### 3.1. Acute Asthma Exacerbations

This protocol was reviewed by the Institutional Review Board at Allegheny Health Network (ANH) and qualified for exempt status with waivers of consent and Health Insurance Portability and Accountability Act (HIPPA) authorization. AHN’s electronic patient visit database was queried to obtain a limited data set of daily acute outpatient visits and ED visits for adults aged 18–64 years residing in Clairton zip code 15025 with discharge diagnoses of asthma exacerbations (J45.901, J45.21, J45.31, J45.41 and J45.51) and status asthmaticus (J45.902, J45.22, J45.32, J45.42, and J45.52) during the study periods. AHN is a regional health system consisting of 14 hospitals, 8 urgent care centers, and over 200 outpatient locations. AHN’s Jefferson Hospital is the only site providing ED care in zip code 15025 and has over 25,000 ED visits annually. Data were obtained for two time periods: the active study period ranged from 24 December 2018 to 28 February 2019 and is referred to as the post-fire period; the comparative study period ranged from 24 December 2017 to 28 February 2018 and is referred to as the pre-fire period. Rate calculations assumed a population size of 9616 adults 18–64 years of age in zip code 15025 based on the most recent American Community Survey (ACS) data from 2019 [34].

### 3.2. Other Data

**Figure 1:**
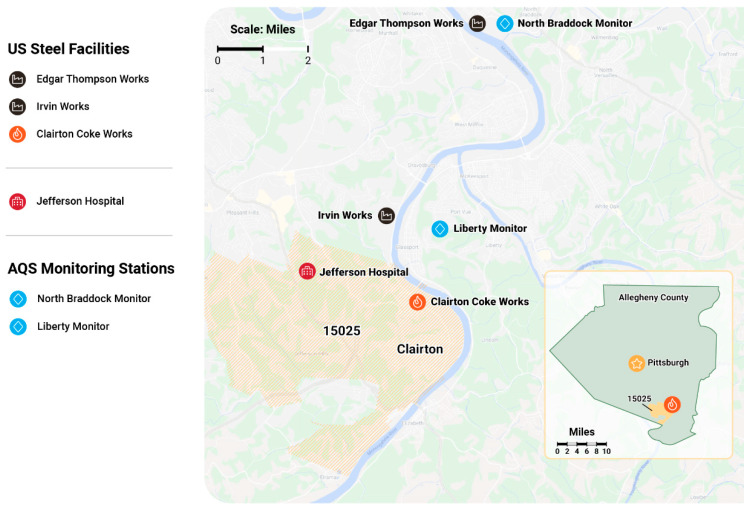
Map of US Steel facilities, AQS monitoring stations and nearest local emergency department. The area of zip code 15025 is shown in tan. The insert in the bottom right corner shows the location of zip code 15025 within Allegheny County, Pennsylvania, US. The prevalent wind direction is from the south–southwest.

Pollution emission data were obtained from a publication of the local county health department and included SO_2_ and H_2_S emissions across three US Steel facilities in the Mon Valley, including Clairton Coke Works, Edgar Thompson Works, and Irvin Works (see Figure 1) [1]. PM_2.5_ emissions from these facilities were not reported. All three facilities were included because coke oven gas was diverted away from the failed desulfurization equipment at Clairton Coke Works and flowed toward the Edgar Thompson and Irvin facilities to be released from flaring stacks into the ambient air. Emission data were expressed as average daily H_2_S grains per hundred dry standard cubic foot (grains/100 dscf) and average daily pounds (lbs/day) of SO_2_. US Steel’s installation permits for all three facilities imposed a site-wide limit for sulfur compound emissions to no more than 35 grains/100 dscf [1].

Ambient air pollution monitoring data were obtained from the Environmental Protection Agency (EPA) website and included PM_2.5_ concentrations obtained from a reference monitor in Liberty and SO_2_ obtained from reference monitors in Liberty and North Braddock (see Figure 1) [35]. These monitoring sites are part of the EPA Air Quality System (AQS) that is used to monitor compliance with the Clean Air Act and were specifically selected since they measured the pollutants of interest, provided temporal data, and were located near and in the wind path of the US Steel facilities. The National Ambient Air Quality Standards (NAAQS), developed by the EPA, are 35 micrograms per meter cubed (ug/m^3^) averaged over a 24-h period for PM_2.5_, and 75 parts per billion (ppb) maximum, hourly, over a 24-h period for SO_2_ [36]. H_2_S data were obtained from the local county health department website and were collected from the reference monitor in Liberty. NAAQS has not developed a standard for H_2_S; however, Pennsylvania has set a standard for H_2_S of 5 ppb averaged over a 24-h period [37].

The National Weather Service (NWS) office in Pittsburgh routinely launches weather balloons (frequently referred to as upper-air soundings) as part of their upper-air observations program. Weather balloons are launched twice each day, at 7 a.m. and 7 p.m. Eastern Standard Time (EST), and collect temperature, humidity, and wind data as they ascend through the atmosphere. Analysis of data collected by weather balloons is used by the NWS to identify the presence and the strength of inversions. The American National Meteorological Society defines inversions as layers in which temperature increases with altitude instead of following the normal pattern of decreasing air temperature with increasing altitude. Inversions can result in stagnant air masses and the accumulation of OAP. On an annual basis, the local county health department summarizes and publishes this data [38]. This study examined all data gathered from the upper-air soundings released by the NWS office in Pittsburgh during the study periods, including average daily temperatures, wind direction and speed, percentage of days with inversions, and strength, depth, and duration of inversions.

The US influenza surveillance system is a collaborative effort between the Centers for Disease Control and Prevention and many partners in state, local, and territorial health departments, public health and clinical laboratories, vital statistics offices, healthcare providers, clinics, and emergency departments [39]. Information is collected from multiple data sources to identify when and where influenza activity is occurring, determine what influenza viruses are circulating, detect changes in influenza viruses, and measure the impact influenza is having on outpatient illness, hospitalizations, and deaths. Influenza data for the 2017–2018 and the 2018–2019 seasons was obtained from the local county health department website [40]. Data included numbers of cases, hospitalizations, and deaths, as well as the peak week of cases each season. A case is defined as testing positive via an antigen, culture, or polymerase chain reaction (PCR) test. Information regarding influenza deaths was received from the Pennsylvania National Electronic Disease Surveillance System (PA-NEDSS) and death certificates. Rate calculations assumed a population size of 753,948 adults 18–64 years of age in the local county based on ACS data reported for 2019 [41].

### 3.3. Data Analysis

Demographic data for Clairton zip code 15025 and Allegheny County were described by distributions for age (<18, 18–64, >65 years), gender (male, female), race (African American, white, other), percentage of persons living below federal poverty level, and median household income. Rates of asthma exacerbation visits by type (acute outpatient or ED) per 1000 residents 18–64 years of age in zip code 15025 were compared in the pre-fire versus post-fire periods using generalized linear model (GLM) analyses with specification of Poisson distribution [42]. This procedure was replicated to assess significance of difference in daily rate of total acute exacerbations on OAP non-exceedance versus exceedance days for PM_2.5_, H_2_S, and SO_2_. Boxplots (median interquartile range [IQR]) were generated to display OAP exposure distributions across all 67 days in each period. Distribution for each OAP metric in the pre-fire versus post-fire periods was assessed for significance of difference using Mann–Whitney U-test due to positive skewing of data. Chi-square test assessed significance in percentage of days in exceedance for SO_2_ (>75 ppb maximum hourly over a 24-h period), H_2_S (>5 ppb averaged over a 24-h period) and PM_2.5_ (>35 ug/m^3^ averaged over a 24-h period) and inversion days that occurred pre-fire versus post-fire. Temperature, wind direction and speed, and strength, depth, and duration of inversions were described by mean (+SD) in each period and compared using the independent t-test. Rates of influenza cases, hospitalizations, and deaths per 1000 residents 18–64 years of age in Allegheny County were compared pre-fire versus post-fire using GLM with Poisson distribution specified as done for asthma exacerbations. Analyses were conducted using SPSS V18.0 and R version 3.6.1.

## 4. Results

### 4.1. Demographics

Due to the limited nature of the data set obtained from AHN, specific demographic information, including race, gender, and age was not available for the study population. However, the demographic composition of residents in Clairton zip code 15025 was similar to the entire local county, as shown Table 1. Approximately 52% of residents were female, 11% lived below the federal poverty level, and race distribution showed 78–80% were white, 13–16% African American, and 4–9% other. Approximately 60% of residents were 18–64 years of age.

### 4.2. Visits for Acute Asthma

**Figure 2:**
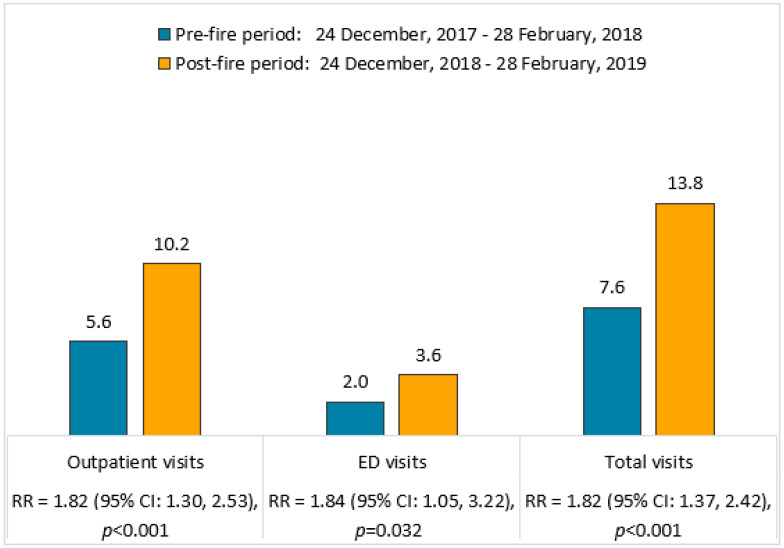
Rates of acute asthma visits by type (outpatient, ED, and total) per 1000 residents (population = 9616 in zip code 15025) before and after the Clairton Coke Works fire. Data were compared using GLM analyses with specification of Poisson distribution. RR = rate ratio.

Figure 2 shows a near doubling of the number of acute outpatient and ED visits for asthma exacerbations in the period after (as compared to the period before) the Clairton Coke Works fire. In the timeframe before the fire, there were 54 acute outpatient visits; and after the fire, there were 98 acute outpatient visits. This translates to a pre-fire versus post-fire increase in the outpatient rate from 5.6 to 10.2 per 1000 residents, respectively (RR = 1.82; 95% CI: 1.30, 2.53; *p* < 0.001). Additionally, in the timeframe before the fire, there were 19 ED visits; and after the fire, there were 35 ED visits. This translates to a pre-fire versus post-fire increase in the ED visit rate from 2.0 to 3.6 per 1000 residents, respectively (RR = 1.84; 95% CI: 1.05, 3.22; *p* = 0.032). Overall, there were 73 and 133 total visits for asthma exacerbations before and after the fire, respectively. The 82% increase from 7.6 to 13.8 total acute asthma visits per 1000 residents pre-fire and post-fire, respectively, was significant (RR = 1.82; 95% CI: 1.37, 2.42; *p* < 0.001).

### 4.3. Outdoor Air Pollution

Table 2 summarizes the total daily average H_2_S and SO_2_ emissions that were released across the three US Steel Mon Valley facilities in the four days prior to and the 36 days following the 24 December 2018 fire at the Clairton Coke Works. After the fire, the total daily average emissions for H_2_S and SO_2_ increased 24 and 35 times, respectively.

**Figure 3:**
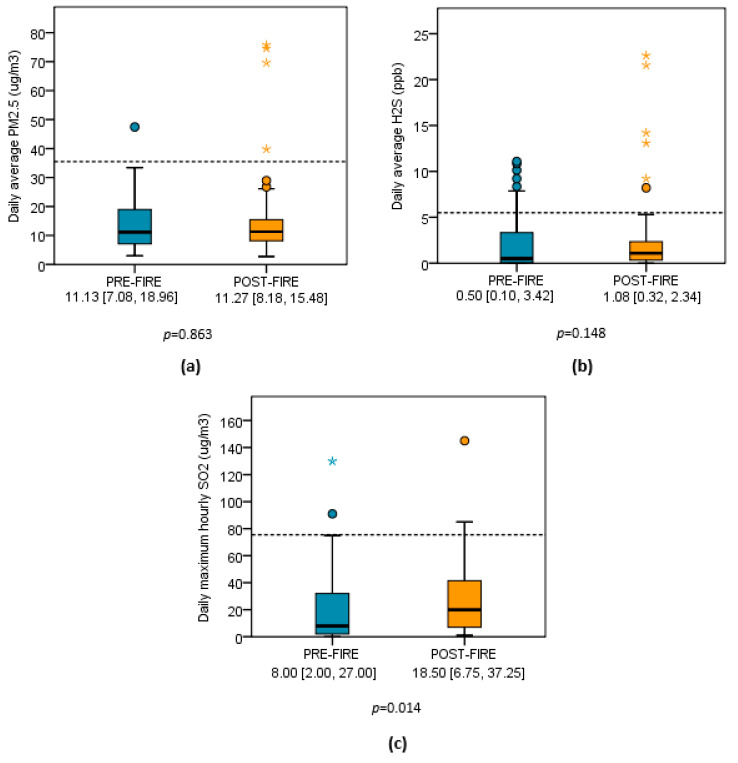
Panels display distributions before and after the Clairton Coke Works fire for (**a**) daily average hourly PM_2.5_, (**b**) daily average hourly H_2_S levels, and (**c**) daily maximum hourly SO_2_. Results are expressed as median (IQR) values. Dashed lines indicate exceedance level for respective OAP exposure: PM_2.5_ >35 µg/m^3^, H_2_S >5 ppb, SO_2_ >75 µg/m^3^. Distributional differences were tested for significance using Mann–Whitney U test due to positive skewing of data.

Figure 3 displays the distribution of daily average PM_2.5_ and H_2_S levels and daily maximum hourly SO_2_ levels that were recorded at monitors in the pre-fire and post-fire periods. Post-fire, the distribution of the median SO_2_ level more than doubled that observed in the pre-fire period. Pre-fire and post-fire median (IQR) SO_2_ (ug/m^3^) distributions were 8.00 (2.00, 27.00) and 18.50 (6.75, 37.25), respectively (*p* = 0.014). Distributions for PM_2.5_ and H_2_S did not significantly differ pre-fire versus post-fire (*p* > 0.05). Pre-fire and post-fire median (IQR) PM_2.5_ (ug/m^3^) distributions were 11.13 (7.08, 18.96) and 11.27 (8.18, 15.48), respectively (*p* = 0.863). Pre-fire and post-fire median (IQR) H_2_S (ppb) distributions were 0.50 (0.10, 3.42) and 1.08 (0.32, 2.34), respectively (*p* = 0.148).

Table 3 summarizes the dates of OAP exceedances that were recorded in the post-fire period at the Liberty and North Braddock reference monitors. During this period, there were four NAAQS PM_2.5_ exceedances (>35 ug/m^3^ averaged over 24 h) at the Liberty monitor, five NAAQS SO_2_ exceedances (>75 ppb averaged over an hour) at the Liberty monitor and two NAAQS SO_2_ exceedance at the North Braddock monitor, and six state H_2_S exceedances (>5 ppb averaged over 24 h) at the Liberty monitor. There were more days with PM_2.5_ and SO_2_ exceedances post-fire as compared to pre-fire. For PM_2.5_, the percentage of days with exceedances were 1.5% pre-fire versus 6.2% post-fire (*p* = 0.161). For SO_2_, the percentage of days with exceedances were 3.0% pre-fire versus 10.4% post-fire (*p* = 0.084). The drastic increase in SO_2_ to 145 ppb measured at the Liberty monitor on 28 December 2018 was attributed to high emissions from the Clairton Coke Works facility in the presence of a weak weather inversion and light winds from the south–southwest direction [38]. There were not more days with H_2_S exceedances post-fire as compared to pre-fire; the percentage of days with H_2_S exceedances were 14.9% pre-fire versus 9.1% post-fire (*p* = 0.301).

**Figure 4:**
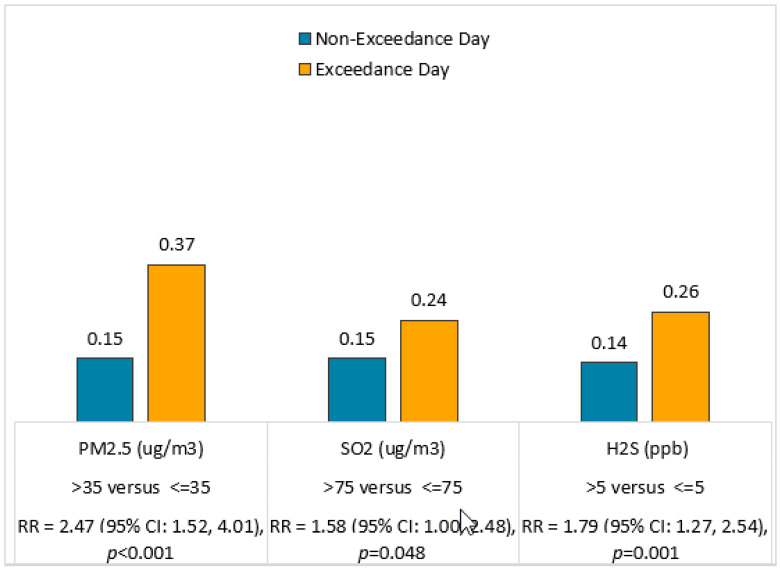
Daily rate of total acute asthma visits per 1000 residents (population = 9616 in zip code 15025) on OAP (PM_2.5_, SO_2_ and H_2_S) non-exceedance and exceedance days. Data were compared using GLM analyses with specification of Poisson distribution. RR = rate ratio.

Figure 4 shows the rate of total outpatient and ED asthma visits (per 1000 residents) on days with (as compared to days without) OAP exceedances during both study periods. The rate of total asthma visits was 0.15 on PM_2.5_ non-exceedance days (<35 ug/m^3^) and more than doubled to 0.37 on PM_2.5_ exceedance days (>35 ug/m^3^). On days with PM_2.5_ >35 versus <35 ug/m^3^, the RR was 2.47 (95% CI: 1.52, 4.01; *p* < 0.001). The rate of total asthma visits was 0.15 on SO_2_ non-exceedance days (<75 ug/m^3^) and increased to 0.24 on exceedance days (>75 ug/m^3^). On days with SO_2_ >75 versus <75 ug/m^3^, the RR was 1.58 (95% CI: 1.00, 2.48; *p* = 0.048). The rate of total asthma visits was 0.14 on H_2_S non-exceedance days (<5 ppb) and increased to 0.26 on H_2_S exceedance days (>5 ppb). On days with H_2_S > 5 versus <5 ppb, the RR was 1.79 (95% CI: 1.27, 2.54; *p* = 0.001).

### 4.4. Weather Data

Table 4 summarizes the weather inversion data during each of the time periods of study. There was no evidence of increased weather inversion events before as compared to after the fire. The number of days with inversions was 24 (35.8%) and 17 (25.3%) pre-fire and post-fire, respectively (*p* = 0.32). The average daily strength, depth, and duration of inversions did not significantly differ between the time periods.

The average daily temperature and wind direction and speed did not differ significantly during the time periods (data not shown). The average (+ SD) daily temperature (°F) was 30.3 + 16.0 and 31.3 + 10.9 (*p* = 0.72) pre-fire and post-fire, respectively. The average (+ SD) daily wind direction (degrees with north = 0, east = 90, south = 180 and west = 270) was 244.3 + 64.3 and 227.5 + 66.4 (*p* = 0.14) pre-fire and post-fire, respectively. The average (+ SD) daily wind speed (miles per hour) was 8.2 + 3.2 and 8.9 + 4.0 (*p* = 0.23) pre-fire and post-fire, respectively.

### 4.5. Influenza Rates

Table 5 summarizes the influenza season data for the local county during each of the time periods of study. There was no evidence that severity or peak of influenza season contributed to the post-fire findings of increased asthma visits. The influenza season was milder post-fire as compared to pre-fire and it peaked outside of the post-fire study period. In fact, in the pre-fire as compared to the post-fire period there were 30% more influenza cases (RR = 1.30; 95% CI: 1.26, 1.33; *p* < 0.05) and 2.8 times more hospitalizations due to influenza (RR = 2.79; 95% CI: 2.44, 3.19; *p* < 0.001).

## 5. Discussion

**Figure 5:**
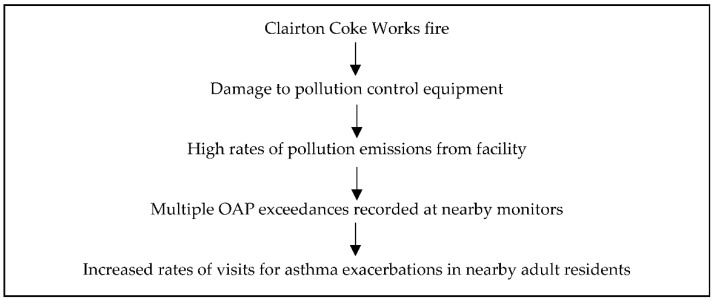
Summary of events related to the industrial incident and subsequent increased rates of visits for asthma exacerbations in nearby adult residents.

This study objectively assessed the impact of a large industrial fire and resultant damage to pollution desulfurization equipment on asthma morbidity in nearby adult residents. Repairs to this equipment were not completed for several months and facility production continued at pre-fire levels during this time-period. Pollution emissions from the facility were significantly increased and multiple OAP exceedance were recorded at local monitors during this period. Figure 5 summarizes the events related to this industrial incident and Table 6 summarizes the study results. The results document a near doubling of the rate of outpatient and ED visits for asthma exacerbations in the months following this incident when damaged pollution control equipment was offline. They also show increased rates of total outpatient and ED visits for asthma exacerbations on days with OAP exceedances. Additionally, the results show that these acute visits were unrelated to confounding factors including weather inversions and seasonal influenza activity. In fact, the influenza season was significantly more severe pre-fire as compared to post-fire. Similarly, weather inversions trended toward being more severe pre-fire as compared to post-fire. The results of this study contribute to the identification and understanding of the effect of this incident on health outcomes and should guide the development of relevant public policies to protect the health of impacted residents during such events.

The results of the current study are consistent with those of a recent retrospective investigation that documented increased self-reported asthma symptoms and rescue medication use in adults with asthma residing near this facility following the Clairton Coke Works fire [6]. In addition to confirming the impact of this incident on asthma morbidity, the current study expands upon these previous findings in several ways. First, the current study used a medically documented discharge diagnosis of asthma exacerbation as an objective measure of asthma morbidity as compared to the subjective outcome of self-reported symptoms and rescue medication used in the prior investigation. Second, the prior investigation reported exclusively on the impact of SO_2_ emissions and monitor exceedances, whereas the current study included assessments of additional relevant OAP constituents, including PM_2.5_ and H_2_S. Third, the current study included an assessment of potential confounding factors including weather inversions and respiratory infections. Finally, the current study analyzed comparative time-periods (pre-fire and post-fire) among the same study population, while the previous investigation analyzed only post-fire outcomes in a nearby impacted population versus a distal non-impacted population. Collectively, the results of these two independent studies show the impact of this industrial incident on multiple indicators of asthma morbidity, including asthma symptoms, rescue medication use, acute outpatient visits, and ED visits.

The results of the current study are also consistent with prior reports documenting associations between exposure to elevated levels of OAP and asthma morbidity. Epidemiologic studies have consistently reported an association between short-term SO_2_ exposures and asthma morbidity, including acute outpatient visits, ED visits, and hospital admissions [9,10,11]. Similarly, other studies have shown an association between short-term PM_2.5_ exposure and these same asthma outcomes [12,13,14]. Fewer studies have examined the association between short-term H_2_S exposure and asthma morbidity. Studies have reported conflicting results, with some showing an association between H_2_S exposure and increased asthma morbidity, while others show no or protective effects of H_2_S on asthma morbidity [43,44,45,46].

Other investigations documented the acute impacts of industrial incidents and associated OAP exposures on respiratory outcomes in nearby residents. A recent study documented the impact of a prolonged coal fire and subsequent PM_2.5_ elevations on asthma morbidity [15]. In that report, the relative risks for daily counts of asthma related ED visits and hospital admissions were 2.32 (95% CI: 1.71, 3.14) and 1.83 (95% CI: 1.14, 2.94), respectively, during the fire period as compared to the non-fire period. Similarly, an earlier study documented the impact of the closure and reopening of a US steel mill on PM_10_ levels and hospital admissions for acute respiratory illnesses including asthma [47]. In that study, pediatric respiratory admissions were two to three times higher when the mill was open as compared to when it was closed. The outcome rates reported in both of those investigations are similar to the increased outcome rates reported in the current study.

The current study did not examine the impact of chronic OAP exposure on asthma outcomes in the study population. However, our group recently documented both high rates of asthma in children residing near point sources of OAP including the U.S. Clairton Coke Works and Edgar Thompson Works facilities [48]. In that study, we found that 70% of participants had exposure to PM_2.5_ greater than the World Health Organization WHO) standard of 10 μg/m^3^. Overall prevalence of asthma was 22.5% (as compared to national rate of 8.3%) with PM_2.5_ and sulfur exposures significantly related to increased odds of asthma. Another recent retrospective study reported decreased historical lung function in adults with asthma residing near the Clairton Coke Works facility who experienced increased symptoms and rescue medication use following the fire [6]. Epidemiologic studies previously documented the strong association between long-term PM_2.5_ exposure and poor asthma outcomes including decreased lung function in both children and adults, decreased lung function growth rate in children, and asthma prevalence [49,50,51].

The current study did not examine the impact of the acute fire and subsequent OAP exceedances on long-term respiratory effects in the exposed population. However, a recent study reported increased lower respiratory symptoms and asthma in nearby residents six years after exposure to high SO_2_ levels following a large sulfur stockpile fire in Africa [52]. Several other studies documented persistence of adverse respiratory effects, including bronchial hyper-responsiveness and asthma, for 3 months to 14 years after the initial acute exposure to high levels of SO_2_ [53,54,55]. Future studies are needed to assess the long-term respiratory effects of the Clairton Coke Works fire and subsequent OAP exceedances in nearby residents.

The results of the current study are important because they objectively document the adverse impact of an industrial incident and subsequent OAP exceedances on health outcomes. Additionally, they emphasize the need for additional public health policies to protect vulnerable residents from future events. Recently, the local health agency has proposed that industries develop strategies to reduce emissions during exceedances of OAP levels. Additional efforts need to focus on developing a rapid alert system to immediately notify impacted residents so they can implement health protective measures during such events. Several countries have already implemented such systems and numerous studies confirm that susceptible populations do modify their behavior to protect their health in response to such alerts [23,24,25,26,27,28,29]. Moreover, a comprehensive rapid response system should be put in place to quickly assess immediate health effects and provide necessary preventative and emergent medical care. Recent literature confirms the need for and success of such systems [30,31,32,33]. Finally, a health registry should be developed in vulnerable communities to track short-term and long-term outcomes related to both chronic and acute OAP exposure. This need is underscored by the recent report of worsening asthma symptoms and increased rescue medication use among patients in an existing asthma registry during the Clairton Coke Works fire and subsequent emission and OAP exceedances [6].

The current study has several strengths. First, it examined the impact of a large coke works fire and subsequent OAP exceedances on acute asthma morbidity in the nearby exposed population. This data was obtained by objective reporting of discharge diagnosis at the time of the visit and did not rely on subject recall or self-report of symptoms and rescue medication use. We controlled for seasonality by using the comparative time-period for the prior year. We also showed that this increase in asthma visits was not due to air stagnation related to weather inversions. This was particularly important given the region’s topography of a river valley surrounded by hills and results of other studies demonstrating increased asthma morbidity during weather inversions [56]. We also showed that the increase in asthma visits was not due to a more severe influenza season. This is important because respiratory viral infections, including influenza, are recognized as triggers of asthma exacerbations. Finally, we used PM_2.5_ and SO_2_ data obtained from relevant, nearby US EPA AQS reference monitors located in the wind path of the industrial sites, and current regulatory thresholds established by the US EPA to assess the impact of these two pollutants on asthma visits. The latter is important because the most recent integrated health assessments by the US EPA concluded the existence of “causal” and “likely to be causal” relationships between short-term SO_2_ and PM_2.5_ exposures, respectively, and respiratory morbidity, particularly in individuals with asthma [57,58].

The main limitation of the current study is that it was conducted using a limited data set and specific demographic data on the asthma patients was not available. We did report that the demographic profile of the Clairton zip code was similar to that of Allegheny County; however, that does not rule out more localized demographic differences. Indeed, the Clairton Coke Works facility is directly adjacent to census tracts that are recognized as environmental justice communities [59]. This is consistent with recent reports, which documented that minorities and those with lower socioeconomic status are much more likely to reside near OAP sources [60,61,62].

The second limitation is that it did not assess the impact of other OAP sources on asthma morbidity. However, at the time of this incident, there were no other reports of increased OAP from relevant sources, including both industrial sites and mobile sources such as traffic. As such, it can be concluded that the OAP exceedances that occurred at the relevant monitors after the industrial incident were attributed to emissions released due to the fire and the resulting breakdown of the desulfurization pollution control equipment.

The third limitation is that we did not have information on individual OAP exposures but instead used OAP data collected from centralized monitoring stations. Of note, both the nearest monitoring station and furthest residence in the study geography were located approximately two linear miles from the site of the Clairton Coke Works. As such, it is possible that individual exposures to OAP were underestimated for most residents living closer than two linear miles from the facility. In support of this possibility, a Canadian study documented clusters of ED visits for asthma among children residing in the same census tracts where two industrial OAP were located [63].

Another limitation relates to the need for caution when interpreting our finding that asthma visits were increased on days with H_2_S exceedances >5 ppb averaged over 24 h. We selected this threshold because it is mandated by the state of PA; however, it was established to protect the environment and not public health. As summarized above, the few studies of the effect of H_2_S exposure on asthma outcomes reported conflicting results [43,44,45,46]. Consequently, there are no current international or US health-based standards for regulating H_2_S levels. The WHO has an air quality guideline of 150 µg/m^3^ (10.6 ppb) H_2_S, averaged over a 24-h period. This guideline is based on the avoidance of eye irritation. Moreover, WHO recommends that H_2_S concentrations not exceed 0.005 ppm (5 ppb; 7 µg/m^3^), over a 30-min period, to avoid substantial complaints about odor [64]. As such, the results of this study should not be interpreted as demonstrating a causal association between H_2_S exceedances >5 ppb averaged over 24 h and increased asthma visits; however, the results do not rule out the possibility that the observed H_2_S exceedances are a marker for the presence of another pollutant that contributed to the observed outcomes.

The final limitation relates to the exclusive focus on asthma morbidity as the study outcome. Although we did not examine the impact of the fire and subsequent OAP exceedances on health care costs, it has been reported that the average cost for an ED visit for asthma is approximately USD 1500 [65]. As such, the total costs related to increased ED visits after the fire is approximately USD 24,000. Additionally, we did not examine the impact of the fire on asthma mortality due to the relatively small population of the study geography and the subsequent possibility of statistical error. However, other studies have reported increased asthma and respiratory mortality with short-term OAP exposure [66,67]. Finally, this study focused exclusively on asthma outcomes. Prior studies have documented an impact of short-term OAP on other respiratory outcomes, including COPD-related ED visits [68,69]. It is possible that COPD and other respiratory related ED visits were also increased after the fire; however, examination of this outcome was beyond the scope of this study.

## 6. Conclusions

In summary, the results of this study document a near doubling in the rate of outpatient and ED visits for asthma exacerbations in the months following the Clairton Coke Works fire when damaged pollution control equipment was offline. They also show increased rates of total outpatient and ED visits for asthma exacerbations on days with OAP exceedances. Additionally, the results show that these acute visits were unrelated to confounding factors including weather inversions and seasonal influenza activity. These results contribute to the identification and understanding of the effect of this incident on health outcomes, and will be disseminated to community residents, leaders, and officials to motivate the development of relevant public health policies to protect impacted residents during such events. As discussed above, such policies should include regulations that industries curtail emissions during exceedances of OAP levels. A rapid alert system should be established to promptly notify impacted residents so they can implement protective health strategies during such events. For example, residents with asthma should receive targeted messages to limit OAP exposure, implement self-management plans and start or increase controller medications. Additionally, more vulnerable residents, such as those with asthma or other pre-existing respiratory conditions, should be advised and/or assisted to relocate immediately. A rapid response system should be developed to quickly assess immediate health impacts and provide both preventative and emergent medical care. Finally, a health registry should be developed in vulnerable communities to track short-term and long-term outcomes related to OAP exposure.

## Figures and Tables

**Table 1:** Demographic characteristics of adult residents in Clairton zip code 15025 and Allegheny County, Pennsylvania, US.

Demographic	Clairton, PA (Zip Code 15025) *N* = 16,298	Allegheny County, PA *N* = 1,216,045
Age, years:		
<18	21%	19%
18–64	*59% (N = 9616)*	*62% (N = 753,948)*
≥65	20%	19%
Gender:		
Male	48%	48%
Female	52%	52%
Race:		
African American	16%	13%
White	80%	78%
Other	4%	9%
Below federal poverty level	11%	11%
Median household income	USD 60,669	USD 64,871

Source: U.S. Census Bureau (2019). American Community Survey 5-year estimates. Retrieved from Census Reporter. Profile page for zip code 15025 and Allegheny County, PA: https://censusreporter.org (last accessed on 8 March 2021).

**Table 2:** Total daily average H_2_S and SO_2_ emissions before and after the Clairton Coke Works fire.

Pollutant	Pre-Fire Daily Average Emissions	Post-Fire Daily Average Emissions
H_2_S (grains/100 dscf/day)	10.91	262.25
SO_2_ (lbs/day)	2118.18	74,099.81

**Table 3:** Summary of dates of OAP exceedances following the Clairton Coke Works fire.

Pollutant	Date	Monitor Location	Level
PM_2.5_	26 December 2018	Liberty	40 ug/m^3^
	02 February 2019	Liberty	70 ug/m^3^
	03 February 2019	Liberty	76 ug/m^3^
	04 February 2019	Liberty	75 ug/m^3^
SO_2_	26 December 2018	Liberty	80 ppb
	28 December 2018	Liberty	145 ppb
	02 January 2019	Liberty	81 ppb
	03 January 2019	Liberty	85 ppb
	07 January 2019	North Braddock	83 ppb
	08 January 2019	Liberty	80 mppb
	04 February 2019	North Braddock	82 ppb
H_2_S	26 December 2018	Liberty	14 ppb
	28 December 2018	Liberty	8 ppb
	08 January 2019	Liberty	9 ppb
	02 February 2019	Liberty	13 ppb
	03 February 2019	Liberty	22 ppb
	04 February 2019	Liberty	22 ppb

**Table 4:** Summary of % days with weather inversions and average daily strength, depth, and duration of inversions before and after the Clairton Coke Works fire.

Parameter	Pre-Fire	Post-Fire	*p*-Value
*N* (% Days)	24 days (35.8%)	17 days (25.3%)	0.317
Strength (Daily AVG + STD)	4.3 + 2.7 °C	3.9 + 2.8 °C	0.648
Depth (Daily AVG + STD)	339 + 272 m	297 + 171	0.578
Duration (Daily AVG + STD)	5.3 + 2.7 h	4.6 + 1.9	0.364

*p*-value based on Chi-square test for % days and independent *t*-test for strength, depth, and duration.

**Table 5:** Influenza cases, hospitalizations, and deaths in Allegheny County among residents 18–64 years of age. (Population = 753,948) before and after the Clairton Coke Works fire.

Parameter	Pre-Fire	Post-Fire	Rate Ratio (95% CI)	*p*-Value
# Cases	12,793	9856	1.30 (1.26, 1.33)	<0.001
# Hospitalizations	803	288	2.79 (2.44, 3.19)	<0.001
# Deaths	31	29	1.07 (0.64, 1.77)	0.796
Peak Week	21–27 January 2018	17–23 January 2019		

*p*-value comparing pre vs. post fire rates based on GLM Poisson regression analyses.

**Table 6:** Summary of results documenting the impact of the Clairton Coke Works fire and subsequent damage to pollution control equipment on medical visits for asthma exacerbations among nearby adult residents.

Study Results
Near doubling of rates of visits for asthma exacerbations in the post-fire period. Increased rates of visits for asthma exacerbations on days with OAP exceedances. These increased visit rates were unrelated to weather inversions and seasonal influenza activity.

## Data Availability

Data are available upon reasonable request by contacting the corresponding author at deborahgentile092465@gmail.com.
